# NudCL2 is an Hsp90 cochaperone to regulate sister chromatid cohesion by stabilizing cohesin subunits

**DOI:** 10.1007/s00018-018-2957-y

**Published:** 2018-10-27

**Authors:** Yuehong Yang, Wei Wang, Min Li, Ya Gao, Wen Zhang, Yuliang Huang, Wei Zhuo, Xiaoyi Yan, Wei Liu, Fangwei Wang, Dingwei Chen, Tianhua Zhou

**Affiliations:** 1 Department of Cell Biology and the Cancer Institute of the Second Affiliated Hospital, Zhejiang University School of Medicine, Hangzhou, 310058 Zhejiang China; 2 Life Sciences Institute and Innovation Center for Cell Signaling Network, Zhejiang University, Hangzhou, 310058 Zhejiang China; 3 Sir Run Run Shaw Hospital, Zhejiang University School of Medicine, Hangzhou, 310020 Zhejiang China; 4 Joint Institute of Genetics and Genomic Medicine between Zhejiang University and University of Toronto, Hangzhou, 310058 Zhejiang China; 5 Collaborative Innovation Center for Diagnosis and Treatment of Infectious Diseases, Hangzhou, 310003 Zhejiang China

**Keywords:** Cochaperone, cohesin, Hsp90, NudCL2, Sister chromatid cohesion

## Abstract

Sister chromatid cohesion plays a key role in ensuring precise chromosome segregation during mitosis, which is mediated by the multisubunit cohesin complex. However, the molecular regulation of cohesin subunits stability remains unclear. Here, we show that NudCL2 (NudC-like protein 2) is essential for the stability of cohesin subunits by regulating Hsp90 ATPase activity in mammalian cells. Depletion of NudCL2 induces mitotic defects and premature sister chromatid separation and destabilizes cohesin subunits that interact with NudCL2. Similar defects are also observed upon inhibition of Hsp90 ATPase activity. Interestingly, ectopic expression of Hsp90 efficiently rescues the protein instability and functional deficiency of cohesin induced by NudCL2 depletion, but not vice versa. Moreover, NudCL2 not only binds to Hsp90, but also significantly modulates Hsp90 ATPase activity and promotes the chaperone function of Hsp90. Taken together, these data suggest that NudCL2 is a previously undescribed Hsp90 cochaperone to modulate sister chromatid cohesion by stabilizing cohesin subunits, providing a hitherto unrecognized mechanism that is crucial for faithful chromosome segregation during mitosis.

**Electronic supplementary material:**

The online version of this article (10.1007/s00018-018-2957-y) contains supplementary material, which is available to authorized users.

## Introduction

During mitosis, the proper segregation of duplicated chromosomes into daughter cells is required for maintaining genome integrity. Sister chromatid cohesion, which is mediated by the highly conserved protein complex cohesin, plays an essential role in chromosome segregation [1]. In mammals, the cohesin complex involved in mitosis is composed of four highly conserved subunits. Two structural maintenance of chromosomes proteins (Smc1α and Smc3) form a heterodimer by interacting their hinge regions. One α-kleisin subunit protein (Rad21) interacts with the head domains of Smc1α and Smc3 and effectively closes the ring. One stromal antigen (SA1 or SA2) directly interacts with Rad21 via its C-terminal region to stabilize the cohesin ring structure [1, 2].

The cohesin complex is dynamically regulated by various factors during cell cycle progression in mammalian cells [1]. In telophase and G1, cohesin is loaded onto chromatin by the Nipbl-Mau2 (Nipped-B like-Mau2) complex, which is counteracted by Wapl (wings-apart like) [3, 4]. During S phase, Smc3 is acetylated by Esco1 (establishment of cohesion protein 1) to enhance the cohesin-DNA interaction and promote the recruitment of Sororin to cohesin. This recruitment displaces Wapl from its binding partner Pds5 (precocious dissociation of sisters protein 5) to further stabilize the cohesin ring that entraps the sister chromatids during S/G2 phase [5, 6]. In early mitosis, phosphorylation of SA2 by Plk1 (polo-like kinase 1) and opening of the cohesin ring by Wapl result in the removal of the majority of cohesin from the chromosome arms [7, 8]. At the same time, the centromeric cohesin is protected by the Sgo1-PP2A (shugoshin 1-protein phosphatase 2A) complex and Haspin (histone H3-associated protein kinase) until metaphase [9, 10]. The cleavage of Rad21 by separase and the deacetylation of Smc3 by histone deacetylase Hdac8 at the metaphase–anaphase transition promote the complete removal of cohesin from chromatin [11–13]. This precise regulation of the cohesin complex ensures the accurate chromosome segregation in cell cycle progression. However, little is known about the regulation of cohesin subunits stability.

Heat shock protein 90 (Hsp90) is a highly conserved and abundant chaperone in eukaryotic cells, functioning as a key regulator of proteostasis by mediating the folding and maturation process of its diverse client proteins [14]. Hsp90 has been well documented to be essential for many cellular activities, including cellular homeostasis, transcriptional regulation, chromatin remodeling, and genomic stability [14]. Multiple components of the DNA repair machinery that regulates genomic stability are reported as Hsp90 clients [15]. Inhibition of Hsp90 leads to the degradation of BRCA2 (breast cancer 2), Rad51, and CHK1 (checkpoint kinase 1) and results in genomic instability. However, it is unclear whether Hsp90 is required for the stability of cohesin subunits.

NudC (nuclear distribution gene C) is a highly conserved protein from yeasts to humans [16]. In the filamentous fungus Aspergillus nidulans, NudC has been demonstrated to be an upstream regulator of NudF (a homolog of human LIS1, a key regulator of cytoplasmic dynein) [17]. We cloned and characterized two mammalian homologs of NudC, NudCL (NudC-like protein) and NudCL2 [18, 19]. NudC and NudCL participate in many biological processes, including cell cycle progression and cell migration [18, 20–22]. Recently, our group has found that NudCL2 is able to stabilize LIS1 by enhancing the interaction between LIS1 and Hsp90 [19]. All the NudC homologs share a conserved core domain of p23 (p23 domain), which modulates Hsp90 ATPase activity to promote the folding of client proteins, and are involved in the regulation of protein stability [18, 19, 21, 23, 24]. However, whether the members of the NudC family are the Hsp90 cochaperones is largely unknown.

In this study, we provide evidence that NudCL2 is required for stabilizing cohesin subunits and ensuring accurate sister chromatid separation during mitosis. Our data reveal that NudCL2 significantly inhibits Hsp90 ATPase activity and enhances its chaperone function. Moreover, ectopic expression of Hsp90 efficiently rescues the cohesion defects in NudCL2-depleted cells. Thus, we propose that NudCL2 acts as an Hsp90 cochaperone to maintain cohesin subunit stability, which is crucial for faithful chromosome segregation in mammalian cells.

## Materials and methods

### Plasmids and small interfering RNAs (siRNAs)

Human *Flag*-*NudCL2, Flag*-NudCL2* (with a silent mutation of three nucleic acids in the RNAi targeting region: ACCTTGAAAAGTGACTGCT), *GST*-*NudCL2, His*-*p23* and yeast *His*-*Hsp90* vectors were constructed previously [19, 21]. Human *Hsp90α* was cloned by RT-PCR and inserted into pcDNA 3.1/Myc-His C (Myc/His-tag vector, Invitrogen). Full-length *NudCL2* was cloned by PCR using *Flag*-*NudCL2* as a template and subcloned into pET-28a (His-tag vector, Novagen) and pcDNA 3.1/Myc-His C. Full-length human *SMC1A, SMC3, RAD21 and STAG2* cloned by RT-PCR were inserted into pFastBac-HT A (His-tag vector, Invitrogen). All of these constructs were confirmed by DNA sequencing.

All siRNAs were synthesized by Genepharma. The sequences of the sense strands of the siRNA duplexes are as follows:

- *LIS1*: 5′-GAACAAGCGAUGCAUGAAGTT-3′ [25]
- *NudCL2*: 5′-ACCUUGAGAAAUAACTGCUTT-3′;
- *NudCL2*-*2*: 5′-CAAGGGCAAACUCUUUGAUTT-3′;
- *Plk1*: 5′-GGGCGGCUUUGCCAAGUGCTT-3′ [8]
- *STAG2*: 5′-GUACGGCAAUGUCAAUAUATT-3′ [26]
- *RAD21*: 5′-AUACCUUCUUGCAGACUGUTT-3′ [8]
- *SMC1A*: 5′- GGAAGAAAGUAGAGACAGATT-3′ [27]
- *SMC3*: 5′-UGUCAUGUUUGUACUGAUATT-3′ [28].

### Cell culture and transfections

HeLa and HEK-293 cells were maintained in Dulbecco’s Modified Eagle’s Medium (DMEM, Corning) supplemented with 10% fetal bovine serum (Invitrogen) at 37 °C in 5% CO_2_. Plasmids were transfected with PolyJet (SignaGen) and the siRNA duplexes were transfected with Lipofectamine RNAiMAX (Invitrogen). The transfection processes were performed according to the manufacturer’s instructions.

### Drug treatments

Geldanamycin (GA, Tocris) was stored at − 20 °C as a stock solution of 1.78 mM in DMSO. Cells were treated with geldanamycin for the indicated times as described in the text. The final concentration of GA was 1.78 μM for HeLa cells and 0.5 μM for HEK-293 cells. For the chromosome spread experiments, 100 ng/ml of colcemid (Sigma) was used to treat cells for 2.5 h.

### Antibodies

For immunofluorescence, antibodies against α-tubulin (Sigma, T6199), cyclin B1 (Santa Cruz, sc-70898), Bub1 (Abcam, ab900), Mad2 (Santa Cruz, sc-28261), Hec1 (Abcam, ab3613) and SMC2 (Abcam, ab10412) and human CREST autoimmune serum (Antibodies, Inc., 15-235-0001) were used. For Western blot analysis, antibodies against Rad21 (Bethyl, A300-080A), SA2 (Bethyl, A300-581A), Smc1α (Bethyl, A300-055A), Smc3 (Bethyl, A300-060A), Mau2 (Abcam, ab46906), Sgo1 (Santa Cruz, sc-54329), Plk1 (Santa Cruz, sc-53418) and Hsp90 (Abcam, ab59459) were utilized. The anti-NudCL2 antibody was generated as described previously [19]. An antibody against actin (Sigma, T1978) was used as the loading control. The secondary antibodies for immunofluorescence analyses were Alexa Fluor 488-conjugated anti-rabbit or anti-mouse IgG and Alexa Fluor 568-conjugated anti-human IgG (Invitrogen). Goat anti-mouse or anti-rabbit secondary antibodies (LI-COR) conjugated with either Alexa Fluor 680 or IRDye 800 were used for Western blot analysis.

### Immunofluorescence staining

HeLa or HEK-293 cells grown on glass coverslips were fixed for 15 min with cold methanol (− 20 °C) and then incubated with primary antibodies for 2 h and secondary antibodies for 1 h at room temperature. DNA was stained with DAPI (Sigma).The mounted coverslips were analyzed by confocal fluorescence microscopy with an oil immersion 60 × objective (LSM510, Zeiss).

### Live-cell images

HeLa cells stably expressing GFP-H2B were treated with either siRNA for 72 h or GA for 48 h. Then, the cells were analyzed with time-lapse video microscopy to track the cell cycle progression. Fluorescence images were captured at 3-min intervals for at least 3 h with LSM510 software (Zeiss). The videos were further edited using ImageJ software (NIH).

### GST pull-down assays

GST pull-down assays were performed as described previously [20, 29]. GST and GST-NudCL2 were purified from bacteria. His-tagged Smc1α, Smc3, Rad21 and SA2 were purified from insect Sf9 cells using the Bac-to-Bac baculovirus expression system (Invitrogen). To detect the association among NudCL2, cohesin subunits and Hsp90, the blots were probed with the respective antibodies as indicated in the text.

### Immunoprecipitation and Western blot assays

Immunoprecipitation for endogenous proteins was performed as described previously [20]. Briefly, whole-cell extracts were generated in TBSN buffer (20 mM Tris [pH 8.0], 150 mM NaCl, 0.5% Nonidet P-40, 5 mM EGTA, 1.5 mM EDTA, 0.5 mM Na_3_VO_4_, 20 mM p-nitrophenyl phosphate) supplemented with protease inhibitors and subjected to coimmunoprecipitation analysis with the indicated antibodies. Western blot analyses were performed with the indicated antibodies and analyzed using the LI-COR Odyssey (LI-COR) system.

### Chromosome spread assays

Chromosome spread assays for Giemsa staining were prepared as previously described [30]. In brief, cells were treated with 100 ng/ml of colcemid (Sigma) for 2.5 h, and mitotic cells were collected by shaking-off. Cells were incubated in a hypotonic solution (DMEM: H_2_O at a ratio of 2:3) for 5.5 min at room temperature and fixed with freshly prepared Carnoy’s solution (75% methanol, 25% acetic acid). Cells in Carnoy’s solution were dropped onto glass slides, stained with 5% Giemsa (Solarbio) and analyzed by bright field microscopy (BX51, Olympus). To perform chromosome spreads for immunofluorescence staining, HeLa cells grown on coverslips were treated with 100 ng/ml of colcemid (Sigma) for 2.5 h to enrich mitotic cells. After washing three times with PBS, the cells were incubated in 75 mM KCl hypotonic solution for 15 min at 37 °C and centrifuged for 5 min at 2000*g* to spread the chromosomes onto coverslips. The samples on coverslips were washed with PHEM buffer (60 mM PIPES, 25 mM HEPES, 10 mM EGTA, 2 mM MgCl_2_, pH 6.9), fixed for 5 min with cold methanol (− 20 °C) and processed for immunofluorescence microscopy (LSM510, Zeiss).

### Hsp90 ATPase assays

Hsp90 ATPase assays were performed as described previously [21]. Briefly, His-Hsp90 (1 µM) was incubated with His-NudCL2 (1 µM), His-p23 (1 µM) or geldanamycin (1.78 µM) at 37 °C for 20 min in reaction buffer (50 mM Tris, pH 7.4, 20 mM KCl, 6 mM MgCl_2_, 1 mM dithiothreitol, 0.5 mM ATP). The released inorganic phosphate was determined by measuring the absorbance at 650 nm using a Cyto Phosphate Assay BIOCHEM kit (Cytoskeleton).

### Aggregation assays

The aggregation reactions of CS (Sigma) or luciferase (Promega) were carried out as described previously [31]. Briefly, CS (0.15 μM) or luciferase (0.15 μM) was incubated alone or with BSA (0.15 μM), NudCL2 (0.15 μM) or Hsp90 (0.15 μM) at 43 °C (for CS) or 42 °C (for luciferase) for 30 min in 40 mM HEPES-KOH (pH 7.5). To monitor the kinetics of thermal aggregation, light scattering was measured at 370 nm by a DU 800 spectrophotometer (Beckman).

### CS thermal inactivation assays

The enzyme inactivation assay of CS was performed as described previously [31, 32]. Briefly, CS (0.15 μM) was incubated at 43 °C in the absence or presence of IgG (1.2 μM), NudCL2 (0.6 μM) or Hsp90 (0.6 μM) in the inactivation buffer (40 mM HEPES-KOH, 0.1 mM EDTA, pH 7.5). Aliquots (100 μl) were taken at the indicated times and mixed with 650 μl of 100 mM Tris (pH 8.1), 50 μl of 3 mM acetyl-CoA (Sigma), 100 μl of 1 mM DTNB (Sigma), and 100 μl of 5 mM oxaloacetate (Sigma), and then incubated at 30 °C for 1 min to eliminate the false readings. To monitor CS activity, the readings were measured at 30 °C for 1 min with 20-s intervals at 412 nm by SpectraMax (Molecular Devices).

### Luciferase refolding assays

Luciferase refolding assays were carried out as described previously [31]. Luciferase (0.2 μM) was incubated either alone or in the presence of IgG (2 μM), NudCL2 (0.2 μM) or Hsp90 (0.2 μM) at 22 °C or 42 °C for 15 min in refolding buffer (5 mM MgCl_2_, 10 mM KCl, 2 mM DTT, 50 mM HEPES-KOH, pH 7.5). After incubation, 10 μl of each mixture was immediately added to a solution containing 18 μl of rabbit reticulocyte lysate (RRL) (Promega) and 2 μl of 0.1 M ATP. During the incubation at 30 °C, the luciferase enzyme activity was measured by SpectraMax (Molecular Devices) using the Luciferase Assay System (Promega) at the indicated times. The tubes were treated with 1 mg/ml BSA for 15 min to prevent luciferase adsorption to the walls.

### Statistical analysis

Data are representative of at least three independent experiments. The mean and standard deviations (SD) were calculated for all quantitative experiments. The Student’s *t* test was used to determine statistically significant differences between groups.

## Results

### Depletion of NudCL2 induces chromosome misalignment

**Fig. 1:**
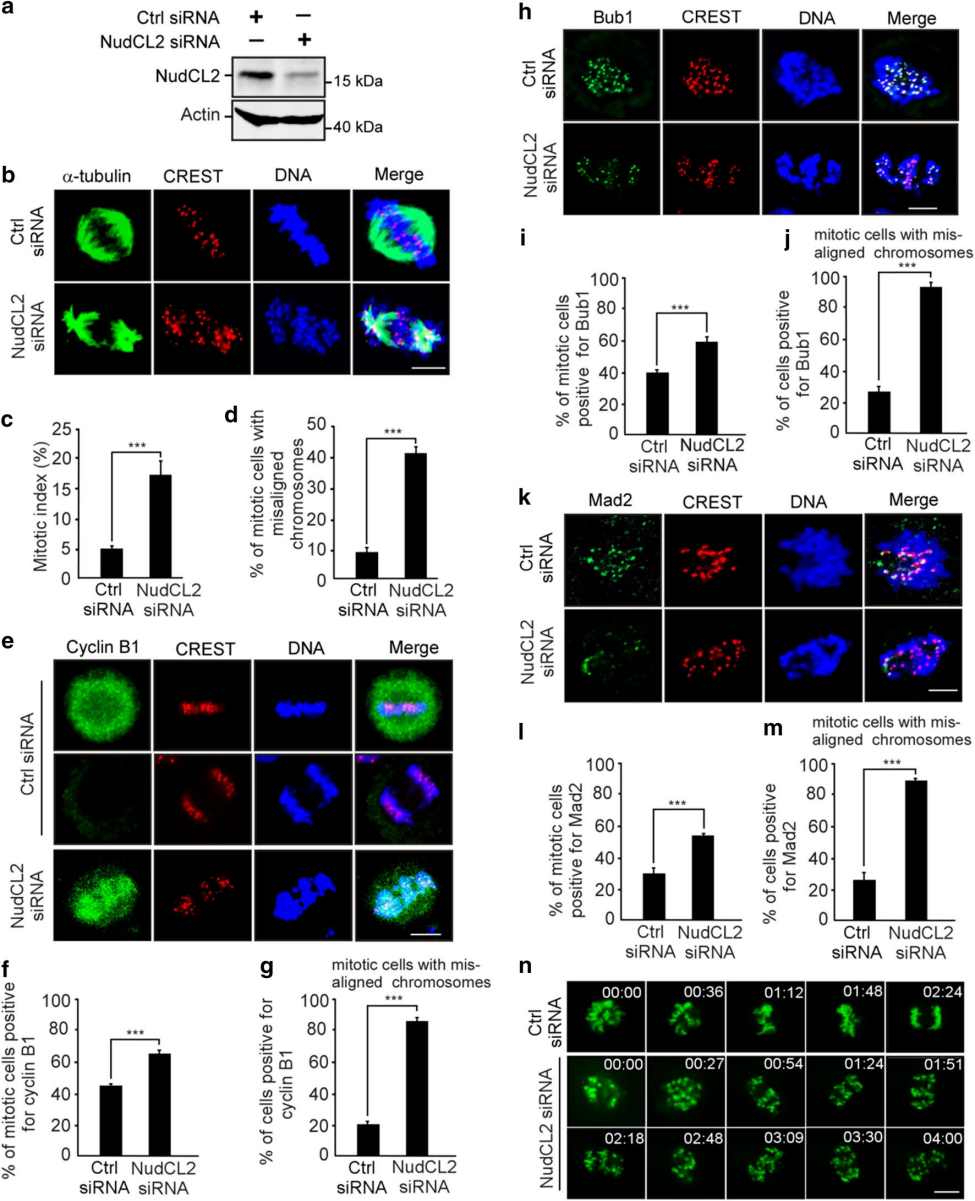
Depletion of NudCL2 causes mitotic defects. HeLa cells were transfected with either control or NudCL2 siRNA for 72 h and subjected to the following analyses: **a** Western blot analysis showed efficient suppression of NudCL2. Actin was used as a loading control. **b**–**d** Immunofluorescence analysis displayed misalignment of chromosomes in NudCL2-depleted cells. Cells were stained with an anti-α-tubulin antibody and human CREST serum (**b**). The mitotic index was calculated (**c**). The percentage of mitotic cells with misaligned chromosomes was significantly higher in NudCL2-depleted cells than in control cells (**d**). **e**–**g** Immunostaining revealed that NudCL2-depleted cells exhibiting misaligned chromosomes were positive for cyclin B1 staining. Cells were probed with an anti-cyclin B1 antibody and human CREST serum (**e**). Mitotic cells positive for cyclin B1 were calculated (**f**). The frequency of cyclin B1-positive mitotic cells with misaligned chromosomes was plotted (**g**). **h**–**m** The spindle checkpoint proteins Bub1 and Mad2 were present at the kinetochores of NudCL2-depleted cells with misaligned chromosomes. Cells were stained with the indicated antibodies and human CREST serum. Mitotic cells positive for Bub1 (**i**) or Mad2 (**l**) were calculated. Mitotic cells with misaligned chromosomes with Bub1 (**j**) or Mad2 (**m**) signals were also counted. **n** Live imaging of HeLa cells stably expressing GFP-H2B showed that some chromosomes failed to congress to the metaphase plate in NudCL2-depleted cells. Time is shown as hh:mm. DNA was visualized with DAPI. Scale bars, 10 μm. Quantitative data are expressed as the mean ± SD (at least three independent experiments). More than 180 cells were counted in each experiment. ***p < 0.001, Student’s *t* test

To address the role of NudCL2 in mitosis, we employed small interfering RNAs (siRNAs) to deplete NudCL2 in HeLa cells. We used two siRNA oligos targeting two different regions of *NudCL2* mRNA (NudCL2 siRNA and NudCL2 siRNA-2). We found that the protein level of NudCL2 was substantially reduced 72 h post-transfection (Fig. 1a; Supplementary Fig. 1a). Immunofluorescence microscopy showed that downregulation of NudCL2 led to the accumulation of mitotic cells (Fig. 1b, c; Supplementary Fig. 1b, c). Further analysis revealed that the percentage of mitotic cells with misaligned chromosomes was significantly higher in NudCL2-depleted cells than in control cells (Fig. 1d and Supplementary Fig. 1d). After ectopic expression of siRNA-resistant NudCL2, approximately 20% of mitotic cells showed misaligned chromosomes compared to less than 10% in the control, and the mitotic index also decreased to about 10% compared to approximately 5% in the control (Supplementary Fig. 2), indicating that the mitotic defects in cells depleted of NudCL2 were partially rescued by ectopic expression of siRNA-resistant NudCL2. In addition, knockdown of NudCL2 in nontumor HEK-293 cells also resulted in mitotic arrest and chromosome misalignment (Supplementary Fig. 3). Collectively, these data suggest that NudCL2 plays an important role in mitosis.

To determine whether the misalignment of chromosomes in NudCL2-depleted cells is due to defects in chromosome congression during prometaphase or chromosome segregation at anaphase, we examined the expression and localization of cyclin B1 and the spindle checkpoint proteins Mad2 and Bub1 in these cells. Approximately, 90% of NudCL2-depleted mitotic cells with misaligned chromosomes were positive for cyclin B1 throughout the cell and were also positive for Mad2 and Bub1 at kinetochores (Fig. 1e–m). Furthermore, live-cell imaging analysis of HeLa cells stably expressing GFP-H2B (GFP-fused histone H2B) revealed that chromosomes failed to align at the metaphase plate in cells depleted of NudCL2 (Fig. 1n, Supplementary Movies. 1, 2). These results strongly indicate that NudCL2 depletion causes defects in chromosome congression during prometaphase, resulting in the misalignment of chromosomes.

### NudCL2 depletion causes premature sister chromatid separation

**Fig. 2:**
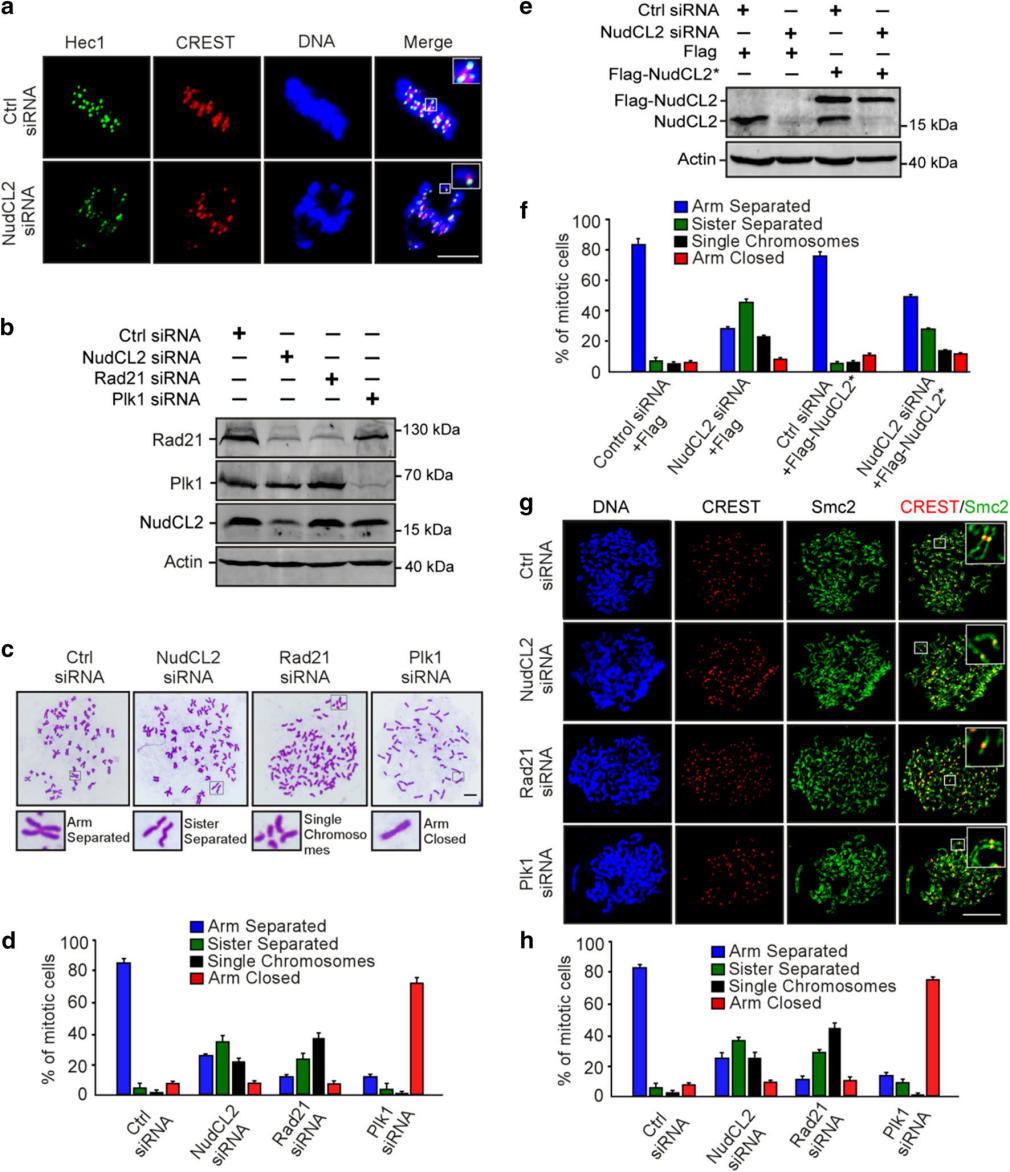
NudCL2 depletion results in premature sister chromatid separation. HeLa cells transfected with the indicated siRNAs and vectors for 72 h were processed for the following analyses: **a** immunofluorescence analysis showed that NudCL2-depleted cells exhibited misaligned chromosomes with one CREST dot associated with only one green Hec1 dot at kinetochores. Cells were stained with an anti-Hec1 antibody and human CREST serum. DNA was visualized by DAPI. **b**–**f** Chromosome spread assays with Giemsa staining revealed precocious sister chromatid separation in cells depleted of NudCL2. Western blot analysis displayed efficient suppression of NudCL2, Rad21 and Plk1 (**b**). Actin was used as a loading control. The cells were treated with colcemid for 2.5 h and subjected to chromosome spread analysis (**c, d**). Ectopic expression of RNAi-resistant NudCL2 (NudCL2*) effectively reversed the defects in sister chromatid separation induced by NudCL2 depletion (**e**, **f**). **g**, **h** Chromosome spread assay followed by immunofluorescence analysis with the indicated antibodies confirmed the precocious separation of sister chromatids in NudCL2-depleted cells. The cells grown on coverslips were treated with colcemid and subjected to chromosome spread and immunofluorescence analysis. DNA was stained with DAPI. Mitotic cells in chromosome spread experiments were categorized into four groups according to their chromosomal morphology: (1) arm separated, where the arms of sister chromatids were separated but connected at centromeres; (2) sister separated (separated sister chromatids), where sister chromatids were separated along the whole chromosome, but their pairing was maintained; (3) single chromosomes, where sister chromatids were completely separated and scattered; (4) arm closed, where sister chromatids were connected at both centromeres and arms. Mitotic cells with different chromosomal morphologies were calculated. Quantitative data are presented as the mean ± SD (at least three independent experiments). More than 150 cells were measured in each experiment. Bars, 10 μm. Higher magnifications of the boxed regions are displayed

To explore how depletion of NudCL2 induces defects in chromosome congression, we first immunostained the centromeric and outer kinetochore markers (CREST and Hec1) on misaligned chromosomes in HeLa cells and found that chromosomes that failed to align at the metaphase plate had one CREST dot associated with only one green Hec1 dot at kinetochores, which is indicative of the loss of sister chromatid cohesion (Fig. 2a). To further investigate the function of NudCL2 in sister chromatid cohesion, we prepared mitotic chromosome spreads in HeLa cells transfected with siRNAs for NudCL2, Rad21 or Plk1. Depletion of the cohesin subunit Rad21 induced cohesion loss as previously described [33], and downregulation of Plk1 stabilized cohesion along chromosome arms [7] (Fig. 2b–d). A higher frequency of mitotic cells displayed separated sister chromatids in NudCL2-depleted cells compared with the control cells, which resembled the Rad21 depletion phenotypes. Similar results were also observed in cells depleted of NudCL2 using another siRNA oligo (Supplementary Fig. 4) and in HEK-293 cells (Supplementary Fig. 5). Furthermore, exogenic expression of siRNA-resistant NudCL2 partially reversed the defects in sister chromatid cohesion in NudCL2-depleted HeLa cells (Fig. 2e, f). Chromosome spreads followed by immunostaining with CREST and Smc2 (a chromosome arm marker) confirmed that NudCL2 depletion led to precocious sister chromatid separation (Fig. 2g, h). Together, these data indicate that NudCL2 plays an essential role in sister chromatid cohesion.

Given that LIS1 is crucial for mitosis and stabilized by NudCL2 [19, 25], we examined whether the role of NudCL2 in sister chromatid cohesion is mediated by LIS1. Chromosome spreads with Giemsa staining clearly revealed that knockdown of LIS1 had no significant effect on sister chromatid cohesion (Supplementary Fig. 6), suggesting that the defects in sister chromatid cohesion induced by NudCL2 depletion are not due to LIS1 downregulation.

### Depletion of NudCL2 destabilizes cohesin subunits

**Fig. 3:**
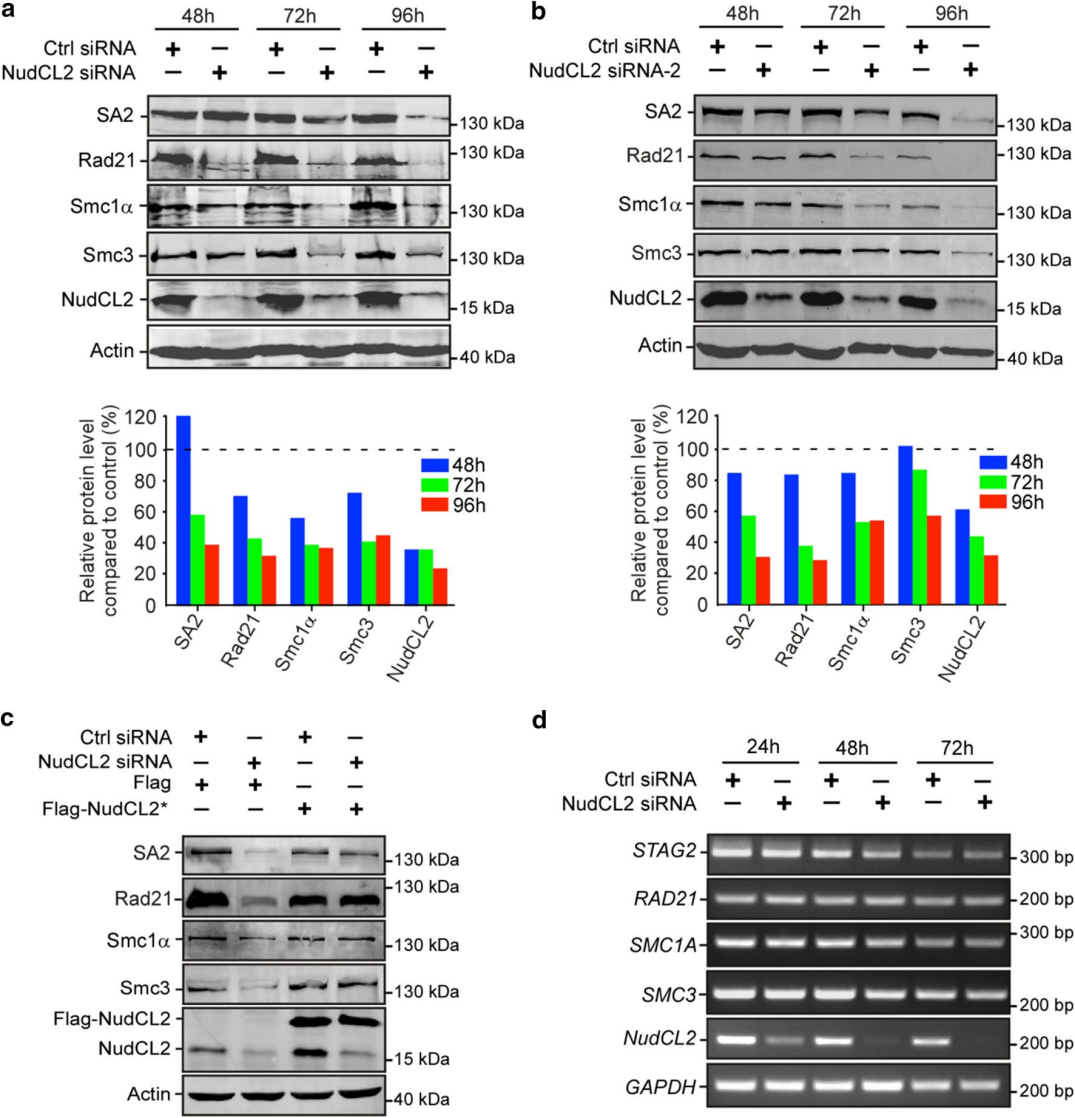
Knockdown of NudCL2 leads to the degradation of cohesin subunits. HeLa cells transfected with the indicated siRNAs and plasmids were subjected to the following analyses: **a**, **b** Western blot analysis with the indicated antibodies revealed that NudCL2 depletion by two different NudCL2 siRNAs effectively decreased the protein levels of cohesin subunits. Actin, a loading control. Relative protein levels compared to the control at the same time point of post-transfection were measured using Image J software and shown at the bottom of each figure. **c** RNAi rescue experiments showed that ectopic expression of NudCL2 rescued the decreased protein levels of cohesin subunits. **d** Semiquantitative RT-PCR demonstrated that knockdown of NudCL2 had no obvious effect on the mRNA levels of cohesin subunits

Since our previous data showed that NudCL2 is involved in the regulation of protein stability [19] and Rad21 protein levels were decreased in NudCL2-depleted cells (Fig. 2b), we next explored whether NudCL2 plays a role in the protein stability of cohesin subunits. Depletion of NudCL2 decreased the protein levels of four cohesin subunits, Smc1α, Smc3, Rad21 and SA2 (Fig. 3a, b). These levels were restored by ectopic expression of siRNA-resistant NudCL2 (Fig. 3c). RT-PCR analysis showed that knockdown of NudCL2 had no effect on the mRNA levels of these subunits (Fig. 3d). It has been reported that downregulation of other cohesin regulatory factors also induces the precocious separation of sister chromatids in mitosis [3, 34]. We examined the protein levels of two crucial cohesin regulatory factors, Mau2 and Sgo1, in cells depleted of NudCL2 and found no changes (Supplementary Fig. 7). Thus, these data strongly indicate that NudCL2 plays an essential role in cohesin subunit stability.

### Inhibition of Hsp90 leads to precocious sister chromatid separation

**Fig. 4:**
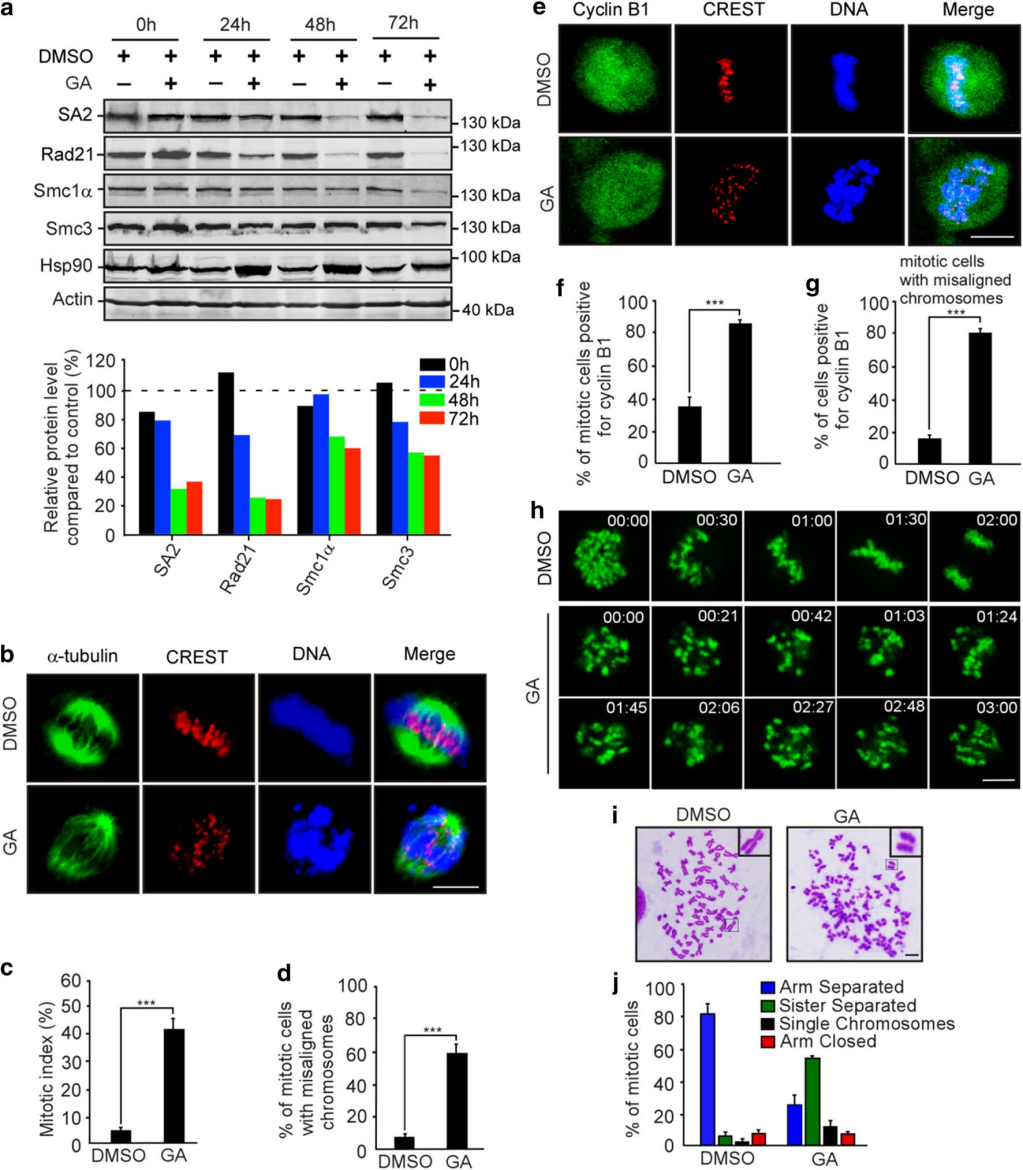
Inhibition of Hsp90 induces defects in sister chromatid cohesion. HeLa cells treated with geldanamycin (GA) or DMSO for 48 h were used for the following analyses: **a** Western analysis with the indicated antibodies revealed that inhibition of Hsp90 by GA treatment obviously reduced the protein levels of cohesin subunits. Actin, a loading control. Relative protein levels compared to the control at the same time point of GA treatment were measured using Image J software and shown at the bottom. **b**–**d** Immunofluorescence showed the misalignment of chromosomes in cells treated with GA. The cells were stained with an anti-α-tubulin antibody and CREST serum (**b**). The mitotic index was calculated (**c**). The percentage of mitotic cells with misaligned chromosomes was plotted (**d**). **e**–**g** Immunostaining displayed that the majority of GA-treated cells with misaligned chromosomes were positive for cyclin B1. Cells were probed with the indicated antibodies (**e**). Cyclin B1-positive mitotic cells were quantified (**f**). The frequency of cyclin B1-positive mitotic cells with misaligned chromosomes was plotted (**g**). **h** Live imaging of HeLa cells stably expressing GFP-H2B showed that chromosomes failed to congress to the metaphase plate in GA-treated cells. DNA was visualized with DAPI. Bars, 10 μm. **i**, **j** Cells treated with GA exhibited premature sister chromatid separation. HeLa cells were subjected to chromosome spreads followed by Giemsa staining after treatment with colcemid for 2.5 h (**i**). Insets, high magnifications of the boxed areas. Mitotic cells with different chromosomal morphologies were counted by the method described in Fig. 2 (**j**). Quantitative data are expressed as the mean ± SD (at least three independent experiments). More than 180 cells were scored in each experiment. ***p < 0.001, Student’s *t* test

Given that NudCL2 facilitates cohesin subunit stability and has a p23 domain that regulates the ATPase activity and chaperoning function of Hsp90 [19, 35], we attempted to assess whether Hsp90 is involved in cohesin subunit stability and sister chromatid cohesion in HeLa cells. Our data revealed that inhibition of Hsp90 ATPase activity by geldanamycin (GA) not only obviously decreased the protein levels of cohesin subunits (Fig. 4a), but also led to a significant increase in the number of mitotic cells with chromosome misalignment that were positive for cyclin B1 staining (Fig. 4b–g). Video microscopy confirmed that inhibition of Hsp90 ATPase activity impaired the alignment of chromosomes at the metaphase plate (Fig. 4h; Supplementary Movies. 3, 4). Further data showed that inhibition of Hsp90 caused premature sister chromatid separation (Fig. 4i, j). Similar results were also observed in HEK-293 cells treated with GA (Supplementary Fig. 8). Together, these data strongly suggest that Hsp90 is critical for the stability of cohesin subunits and sister chromatid cohesion.

### NudCL2 regulates cohesin subunit stability via Hsp90

**Fig. 5:**
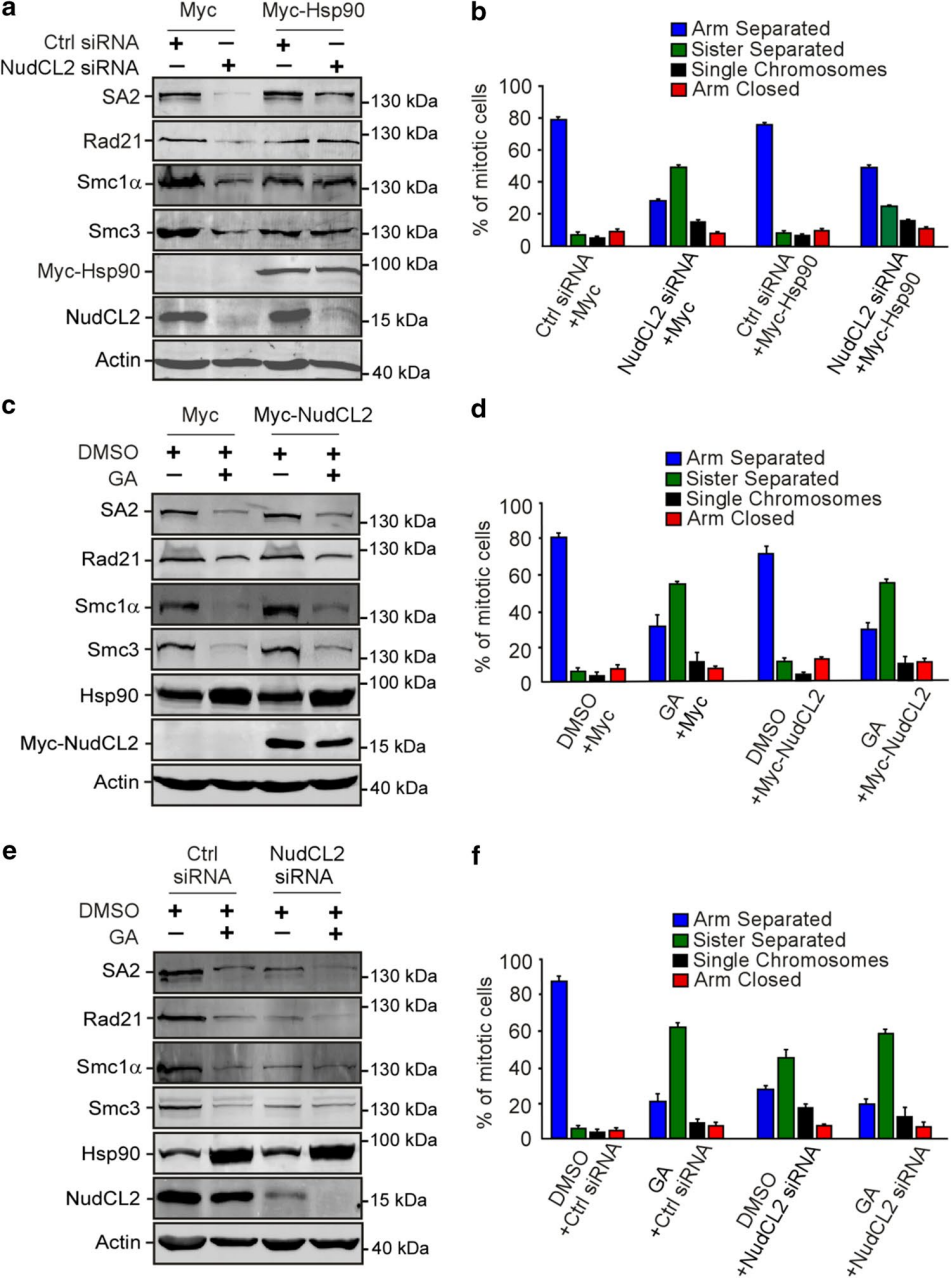
Ectopic expression of Hsp90 rescues the defects caused by NudCL2 depletion but not vice versa. HeLa cells transfected with the indicated siRNAs and vectors were treated with geldanamycin and subjected to Western blotting and chromosome spreads followed by Giemsa staining. Actin was used as a loading control. The percentages of mitotic cells with different chromosomal morphologies were measured by the method described in Fig. 2. Quantitative data are shown as the mean ± SD (at least three independent experiments). More than 170 cells were calculated in each experiment. **a**, **b** Exogenous expression of Hsp90 reversed the degradation of cohesin subunits and premature sister chromatid segregation in NudCL2-depleted cells. **c**, **d** Ectopic expression of NudCL2 was unable to rescue the decreased levels of cohesin subunits and defects in sister chromatid cohesion in cells with Hsp90 inhibition. **e**, **f** Depletion of NudCL2 had no synergistic effects with Hsp90 inhibition on cohesin subunit stability and sister chromatid cohesion

Given that depletion of NudCL2 or inhibition of Hsp90 ATPase activity destabilized cohesin subunits and caused precocious sister chromatid separation (Figs. 2, 3, 4), we speculated that NudCL2 might be involved in the regulation of cohesin stability via Hsp90. We designed a series of rescue experiments and found that the exogenous expression of Hsp90 efficiently reversed the instability of cohesin subunits and the defects in sister chromatid cohesion induced by NudCL2 depletion (Fig. 5a, b). By contrast, ectopic expression of NudCL2 failed to reverse the degradation of cohesin subunits and the defects in sister chromatid cohesion caused by Hsp90 inhibition (Fig. 5c, d). Furthermore, depletion of NudCL2 had no synergistic effect with Hsp90 inhibition on cohesin subunits stability or sister chromatid cohesion (Fig. 5e, f). Thus, these data imply that NudCL2 modulates the stability of cohesin subunits via Hsp90.

### NudCL2 interacts with Hsp90 and cohesin subunits

**Fig. 6:**
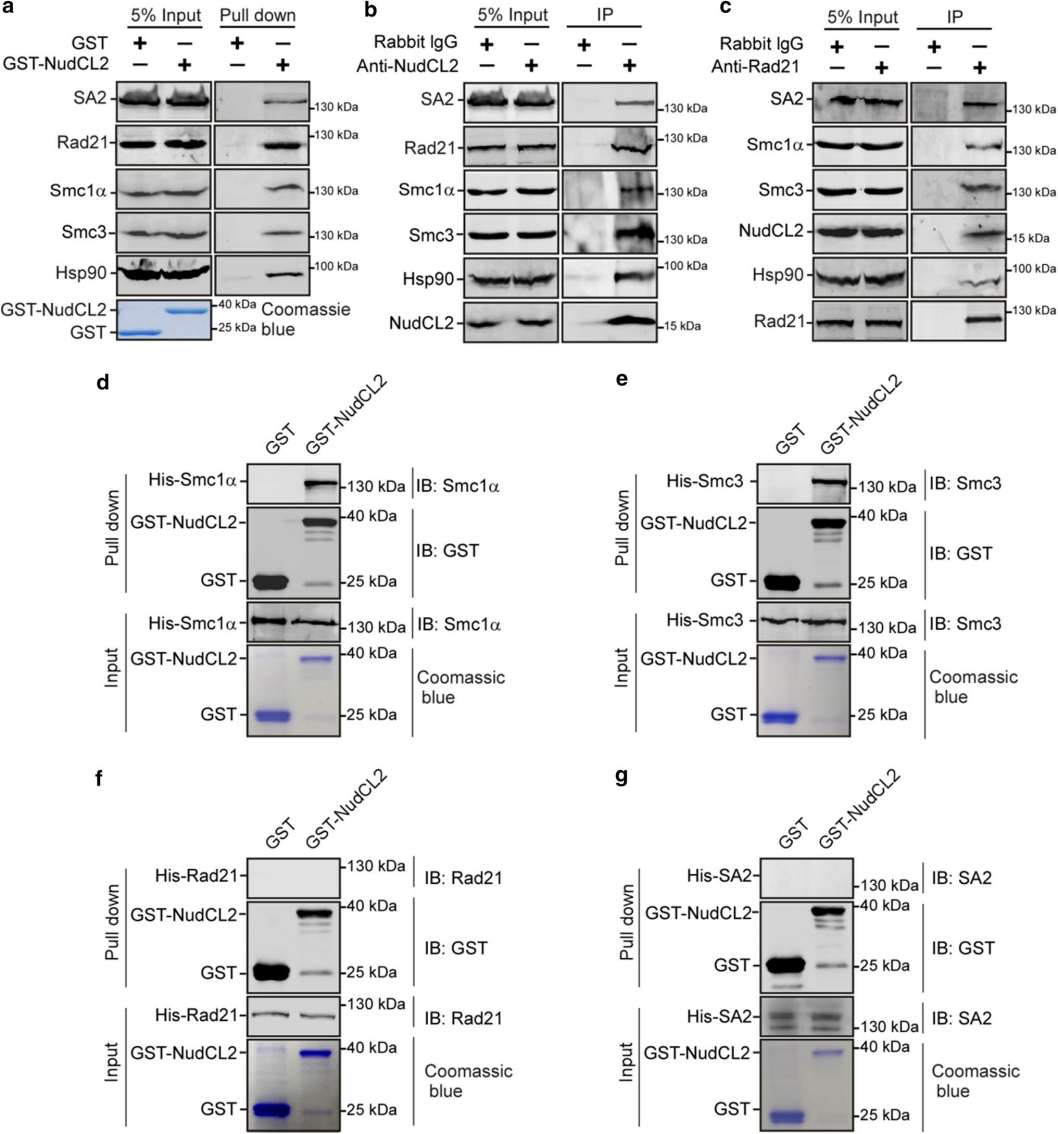
NudCL2 interacts with cohesin subunits and Hsp90 in vitro and in vivo. **a** GST pull-down assays showed that NudCL2 was associated with cohesin subunits and Hsp90 in vitro. Purified GST or GST-NudCL2 protein was incubated with HeLa cell lysates and subjected to immunoblotting. The inputs of GST and GST-NudCL2 were stained with Coomassie brilliant blue. **b**, **c** Endogenous NudCL2 bound to four cohesin subunits and Hsp90 in vivo. Total lysates of HeLa cells were immunoprecipitated with the indicated antibodies or IgGs and processed for Western blotting. **d**–**g** NudCL2 protein directly interacted with Smc1α and Smc3 but not Rad21 or SA2 in vitro. His-tagged Rad21, SA2, Smc1α and Smc3 were expressed in Sf9 insect cells and purified with nickel-nitrilotriacetic acid beads. GST or GST-NudCL2 protein was incubated with each of the purified cohesin subunits and subjected to Western analysis with the indicated antibodies

To investigate how NudCL2 regulates cohesin subunits stability by Hsp90, we first examined the potential interaction among NudCL2, Hsp90 and cohesin subunits. GST pull-down assays using cell lysates revealed that GST-NudCL2 bound to Hsp90 and cohesin subunits (Fig. 6a). Further immunoprecipitation experiments with anti-NudCL2 or Rad21 antibody showed the association of endogenous NudCL2, Hsp90 and cohesin subunits in vivo (Fig. 6b, c). To determine whether NudCL2 directly interacted with cohesin subunits, we purified recombinant cohesin subunits (His-Smc1α, His-Smc3, His-Rad21 and His-SA2) from insect Sf9 cells using the Bac-to-Bac baculovirus expression system. The data showed that GST-NudCL2 protein directly interacted with Smc1α and Smc3, but not Rad21 and SA2 in vitro (Fig. 6d–g).

### NudCL2 functions as an Hsp90 cochaperone

**Fig. 7:**
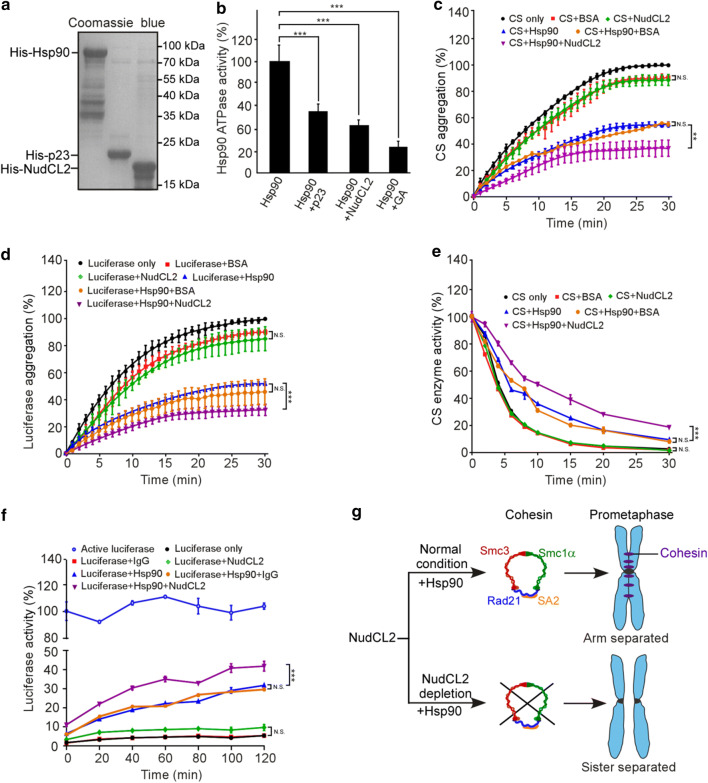
NudCL2 enhances the chaperone activity of Hsp90. **a** Purified His-tagged p23, Hsp90, and NudCL2 proteins were subjected to SDS-PAGE with Coomassie blue staining. **b** NudCL2 inhibited the in vitro ATPase activity of Hsp90. Hsp90 ATPase activity was measured in the presence of p23, NudCL2 or geldanamycin and presented as the activity relative to that of only Hsp90. **c**, **d** NudCL2 suppressed the heat-induced aggregation of citrate synthase (CS) and luciferase with Hsp90. CS (**c**) and luciferase (**d**) were incubated with the indicated proteins for different times. Aggregation was determined by light scattering (370 nm) at each time point and calculated as the percentage of CS or luciferase aggregation after a 30 min incubation. **e** NudCL2 reduced the rate of heat-induced CS inactivation with Hsp90. CS was incubated with the indicated proteins at 43 °C for different times. The enzymatic activity of CS was detected at 30 °C by light scattering at 412 nm. All activities were expressed as a percentage of the initial activity of CS (0 min). **f** NudCL2 facilitated the refolding of luciferase after heat-induced aggregation. Luciferase was incubated at 42 °C for 15 min in the presence of the indicated proteins. The enzymatic activity of luciferase was monitored by a luminometer at each time point. The activity was calculated as a percentage of the activity of luciferase at 22 °C after a 15 min incubation. Quantitative data are presented as the mean ± SD (at least three independent experiments). ***p < 0.001, Student’s *t* test. **g** Working model for the role of NudCL2 in sister chromatid cohesion. NudCL2 stabilizes cohesin subunits by acting as an Hsp90 cochaperone to enhance cohesin stability. Depletion of NudCL2 induces cohesin subunits degradation and results in premature sister chromatid segregation during mitosis

Since NudCL2 not only regulates the stability of cohesin subunits and LIS1, but also contains a conserved p23 domain, the core structure of p23 required for its cochaperone function of Hsp90 [19, 35], we explored whether NudCL2 functions as an Hsp90 cochaperone to stabilize client proteins. We first purified NudCL2, p23 and Hsp90 proteins, and then performed in vitro Hsp90 ATPase assays (Fig. 7a). The results revealed that both NudCL2 and p23 significantly inhibited the ATPase activity of yeast Hsp90 (Fig. 7b). Further heat-induced aggregation experiments with two Hsp90 substrates, citrate synthase (CS) and luciferase, showed that NudCL2 enhanced the chaperone function of Hsp90 to inhibit the aggregation of CS and luciferase, whereas NudCL2 itself did not suppress their heat-induced aggregation (Fig. 7c, d).

Both CS and luciferase enzymes are active in their native state and lose activity upon unfolding or denaturation and this property has been utilized to study the chaperoning function of Hsp90 influenced by its cochaperones [36, 37]. To confirm whether NudCL2 acts as an Hsp90 cochaperone to regulate the enzymatic activity of Hsp90 client proteins, we carried out CS thermal inactivation and luciferase refolding assays in vitro. The results showed that upon Hsp90, NudCL2 not only significantly reduced the heat-induced CS inactivation (Fig. 7e), but also promoted the luciferase activity recovery after heat-induced inactivation (Fig. 7f). Interestingly, in the absence of Hsp90, NudCL2 did not suppress heat-induced CS inactivation or enhance the recovery of luciferase activity after heat-induced inactivation (Fig. 7e, f). Taken all together, these data indicate that NudCL2 may function as an Hsp90 cochaperone to enhance the stability and function of client proteins.

## Discussion

The Hsp90 cochaperone p23 plays a critical role in the stabilization of client proteins, including steroid hormone receptors and telomerase, by inhibiting Hsp90 ATPase activity and stabilizing the ATP-induced conformational change of Hsp90 [35, 38, 39]. Knockdown of p23 destabilizes a number of Hsp90 client proteins [40, 41]. Here, we provide evidence that NudCL2 interacts with Hsp90 and suppresses the ATPase activity of Hsp90 (Figs. 6a–c, 7a, b). Further results reveal that NudCL2 inhibits the heat-induced aggregation and enzymatic inactivation of Hsp90 substrates (Fig. 7c–f). Moreover, we also show that either NudCL2 depletion or Hsp90 inhibition by GA decreases the stability of cohesin subunits and induces premature sister chromatid separation (Figs. 2, 3, 4). Ectopic expression of Hsp90 efficiently rescues the defects induced by NudCL2 depletion, but not vice versa (Fig. 5). These results indicate that the p23 domain-containing protein NudCL2 is an Hsp90 cochaperone to promote its chaperone function and stabilize cohesin subunits (Fig. 7g).

In this study, we find that NudCL2 knockdown and GA’s inhibition of Hsp90’s ATPase cause similar defects (Figs. 2, 3, 4). GA is a natural compound which competes with ATP to bind with high affinity into the ATP binding pocket of Hsp90 and thereby inhibits the ATPase activity of Hsp90 [42]. When cells are treated with GA, Hsp90 loses the ability to fold and activate its client proteins, which results in the degradation of client proteins [43], including cohesin subunits (Fig. 4). Therefore, although NudCL2 functions as a cochaperone to inhibit Hsp90’s ATPase activity, its knockdown and GA treatment induce similar effects.

NudC was first identified in Aspergillus nidulans as a key upstream regulator of the LIS1/dynein complex [17, 44, 45]. Our group and others have found that vertebrate NudC has three principal homologs, NudC, NudCL and NudCL2, all of which contain the conserved p23 domain [18, 19, 21, 23, 24]. NudC is not only able to interact with Hsp90 and regulate LIS1 stability via the Hsp90 pathway, but also stabilizes cofilin 1 to regulate ciliogenesis in an Hsp90-independent manner [21, 24]. Additionally, NudCL has been documented to play an important role in the stability regulation of the dynein intermediate chain [18]. Recently, our data have shown that NudCL2 promotes LIS1 stability by enhancing the interaction between Hsp90 and LIS1 [19]. In this report, we further find that NudCL2 acts as an Hsp90 cochaperone to stabilize cohesin subunits by modulating the ATPase activity of Hsp90. Intriguingly, NudC and NudCL, but not NudCL2, possess an intrinsic chaperoning activity independent of Hsp90 to regulate the stability of client proteins [18, 23, 24]. Taken together, these data imply that the members of NudC family may act as a new type of regulator of protein stability possibly via the Hsp90-dependent or -independent pathway.

Emerging studies have demonstrated the molecular regulation underlying the stability of the cohesin-DNA interaction [4–7, 11]. However, how the stability of cohesin subunits is regulated remains unclear. Recent studies have shown that Smc3 knockdown leads to Smc1α instability, but Smc1α depletion has no significant effect on Smc3 stability [46]. Downregulation of either Smc1α or Smc3 causes Rad21 degradation, while depletion of Rad21 only decreases SA protein levels [46, 47]. Here, our data reveal that knockdown of either Smc3 or Smc1α reduces the stability of the other three cohesin subunits (Supplementary Fig. 9). Rad21 depletion decreases the protein levels of SA2, but not Smc1α or Smc3. SA2 depletion does not affect the stability of the other three subunits. These results indicate that either Smc1α or Smc3 is essential for the stability of Rad21 and SA2, but not vice versa. Moreover, we show that depletion of NudCL2 decreases the protein levels of all cohesin subunits (Fig. 3). Purified GST-NudCL2 directly interacts with His-tagged Smc1α and Smc3, but not Rad21 or SA2 (Fig. 6d–g). Together, these data suggest that NudCL2 may promote the stability of cohesin subunits by directly interacting with Smc1α and Smc3.

In summary, this study provides evidence that NudCL2 is essential for sister chromatid cohesion by acting as an Hsp90 cochaperone to stabilize cohesin subunits. Depletion of NudCL2 causes cohesin subunits degradation and results in premature sister chromatid segregation during mitosis. This work reveals a previously uncharacterized mechanism for the regulation of chromosome segregation in mammalian cells.

### Electronic supplementary material

Below is the link to the electronic supplementary material.

Supplementary material 1 (DOCX 4055 kb)

Supplementary material 2 (AVI 168 kb)

Supplementary material 3 (AVI 167 kb)

Supplementary material 4 (AVI 111 kb)

Supplementary material 5 (AVI 114 kb)

## Abbreviations

BSA: Bovine serum albumin
BRCA2: Breast cancer 2
CHK1: Checkpoint kinase 1
CS: Citrate synthase
Esco1: Establishment of cohesion protein 1
GA: Geldanamycin
GFP-H2B: GFP-fused histone H2B
Haspin: Histone H3-associated protein kinase
Hsp90: Heat shock protein 90
LIS1: Lissencephaly 1 gene product
NudC: Nuclear distribution gene C
NudCL: NudC-like protein
NudCL2: NudC-like protein 2
SD: Standard deviations
Sgo1-PP2A: Shugoshin 1 and protein phosphatase 2A
siRNAs: Small interfering RNAs
Wapl: Wings-apart like
