# Supplementary material for: NudCL2 is an Hsp90 cochaperone to regulate sister chromatid cohesion by stabilizing cohesin subunits
Source: Cell Mol Life Sci. 2018 Oct 27;76(2):381–95. doi: 10.1007/s00018-018-2957-y (PMC6339671; doi:10.1007/s00018-018-2957-y)
Supplement: Supplementary file 1 — Supplementary material 1 (DOCX 4055 kb) [file 18_2018_2957_MOESM1_ESM.docx]

**Supplementary information**

**Supplementary Fig. 1** NudCL2 depletion induces mitotic defects in HeLa cells. HeLa cells were transfected with control or NudCL2 siRNA-2 for 72 h and processed for the following experiments: **a** Western blot analysis showed depletion of NudCL2. Actin was used as a loading control. **b**-**d** Immunofluorescence of NudCL2-depleted cells exhibited chromosome misalignment. Cells were stained with the indicated antibodies (**b**). DNA was labeled with DAPI. Scale bars, 10 μm. Mitotic cells were counted and the mitotic index was calculated (**c**). The percentage of mitotic cells with misaligned chromosomes was higher in NudCL2-depleted cells than in control cells (**d**). Quantitative data are expressed as the mean ± SD (at least three independent experiments). More than 200 cells were counted in each experiment. ***p* < 0.01; ****p* < 0.001, Student’s *t* test.

**Supplementary Fig. 2** Ectopic expression of RNAi-resistant NudCL2 reverses mitotic defects induced by NudCL2 depletion. HeLa cells transfected with the indicated siRNAs and vectors were subjected to the following analyses: **a** Western analysis showed efficient suppression of endogenous NudCL2 and ectopic expression of NudCL2. Actin, a loading control. **b**-**d** Immunofluorescence revealed that ectopic expression of RNAi-resistant NudCL2 (NudCL2*) significantly reversed the mitotic defects caused by NudCL2 depletion. Cells were stained with the indicated antibodies (**b**). DNA was labeled with DAPI. The mitotic index was calculated (**c**). The percentage of mitotic cells with misaligned chromosomes was measured (**d**). Quantitative data are expressed as the mean ± SD (at least three independent experiments). More than 150 cells were counted in each experiment. **p* < 0.05; ***p* < 0.01; ****p* < 0.001, Student’s *t* test.

**Supplementary Fig. 3** NudCL2 depletion induces mitotic defects in HEK-293 cells. HEK-293 cells were transfected with control or NudCL2 siRNA for 72 h and subjected to the following analyses. **a** Western blotting showed obvious depletion of NudCL2. Actin was used as a loading control. **b**-**d** Immunofluorescence analysis of NudCL2-depleted cells exhibited misalignment of chromosomes. Cells were stained with the indicated antibodies (**b**). DNA was visualized by DAPI. The mitotic index was calculated (**c**). The percentage of mitotic cells with misaligned chromosomes was higher in NudCL2-depleted cells than in control cells (**d**). Quantitative data are presented as the mean ± SD (at least three independent experiments). More than 200 cells were calculated in each experiment. ****p* < 0.001, Student’s *t* test.

**Supplementary Fig. 4** NudCL2 depletion results in premature sister chromatid separation in HeLa cells. HeLa cells were transfected with control or NudCL2 siRNA-2 for 72 h and subjected to the following analyses: **a** Immunoblotting showed substantial decrease of NudCL2. Actin, a loading control. **b**, **c** Depletion of NudCL2 induced precocious sister chromatid separation. The cells were treated with colcemid for 2.5 h and subjected to chromosome spreads and Giemsa staining. Representative images are shown (**b**). Insets, high magnifications of the boxed areas. The frequencies of four chromosomal morphologies were measured using the method described in Fig. 2 (**c**). Quantitative data are expressed as the mean ± SD (at least three independent experiments). More than 200 cells were scored in each experiment.

**Supplementary Fig. 5** Depletion of NudCL2 promotes premature sister chromatid separation in HEK-293 cells. HEK-293 cells were transfected with control or NudCL2 siRNA for 72 h and subjected to the following analyses: **a** Immunoblotting displayed efficient suppression of NudCL2. Actin was used as a loading control. **b**, **c** Depletion of NudCL2 led to precocious sister chromatid separation**.** The cells were treated with colcemid and prepared for chromosome spreads followed by Giemsa staining (**b**). Representative images are shown. Insets, high magnifications of the boxed areas. The percentage of cells with different chromosomal morphologies was measured using the method described in Fig. 2 (**c**). Quantitative data are expressed as the mean ± SD (at least three independent experiments). More than 200 cells were measured in each experiment.

**Supplementary Fig. 6** Depletion of LIS1 has no obvious effect on sister chromatid cohesion. HeLa cells were transfected with control or LIS1 siRNA for 72 h and subjected to the following analyses: **a** Western analysis showed efficient suppression of LIS1. Actin was used as a loading control. **b**, **c** Chromosome spreads followed by Giemsa staining revealed that cells depleted of LIS1 displayed no obvious deficiencies in sister chromatid cohesion. Representative images are shown (**b**). Insets, high magnifications of the boxed areas. The frequencies of four chromosomal morphologies were measured by the method described in Fig 2 (**c**). Quantitative data are expressed as the mean ± SD (at least three independent experiments). More than 200 cells were scored in each experiment.

**Supplementary Fig. 7** Knockdown of NudCL2 does not affect the protein levels of Mau2 and Sgo1. HeLa cells were transfected with control or NudCL2 siRNA for the indicated times and subjected to Western blotting with the indicated antibodies. **a**, **b** Depletion of NudCL2 had no effect on the protein levels of Mau2 (**a**) and Sgo1 (**b**). Actin, a loading control.

**Supplementary Fig. 8** Inhibition of Hsp90 causes defects in sister chromatid cohesion in HEK-293 cells. HEK-293 cells treated with GA or DMSO were subjected to the following analyses: **a** Inhibition of Hsp90 decreased the levels of cohesin subunits. The cells treated with GA or DMSO were harvested at the indicated times and subjected to immunoblotting analysis with the antibodies as shown. Actin, a loading control. Relative protein levels compared to the control at the same time point of GA treatment were measured using Image J software and shown at the bottom. **b**-**d** Immunofluorescence showed obvious chromosome misalignment in cells treated with GA for 48 h. Cells were stained with the indicated antibodies (**b**). The mitotic index was calculated (**c**). The percentage of mitotic cells with misaligned chromosomes was calculated (**d**). **e**, **f** Inhibition of Hsp90 caused premature sister chromatid separation. The cells were treated with colcemid for 2.5 h and subjected to chromosome spreads and Giemsa staining (**e**). Representative images are shown. Insets, high magnifications of the boxed areas. The percentages of cells with different chromosomal morphologies were determined as described in Fig 2 (**f)**. Quantitative data are presented as the mean ± SD (at least three independent experiments). More than 200 cells were scored in each experiment. ****p* < 0.001, Student’s *t* test.

**Supplementary Fig. 9** The interdependence of cohesin subunits stability. HeLa cells were transfected with the control or the indicated siRNAs and subjected to Western blot analysis. **a** Smc1α depletion decreased the stability of Smc3, Rad21 and SA2. **b** Knockdown of Smc3 reduced the protein levels of Smc1α, Rad21 and SA2. **c** Downregulation of Rad21 caused a decrease in the protein level of SA2, but not Smc1α or Smc3. **d** Depletion of SA2 had no effect on the other cohesin subunits. Actin, a loading control.

**[Supplementary](https://static-content.springer.com/esm/art%3A10.1038%2Fs41467-018-04849-7/MediaObjects/41467_2018_4849_MOESM3_ESM.avi) Movie 1** Mitotic progression of control cell stably expressing GFP-H2B.

[**Supplementary**](https://static-content.springer.com/esm/art%3A10.1038%2Fs41467-018-04849-7/MediaObjects/41467_2018_4849_MOESM3_ESM.avi) **Movie 2** Mitotic progression of NudCL2-depleted cell stably expressing GFP-H2B.

[**Supplementary**](https://static-content.springer.com/esm/art%3A10.1038%2Fs41467-018-04849-7/MediaObjects/41467_2018_4849_MOESM3_ESM.avi) **Movie 3** Mitotic progression of DMSO-treated HeLa cell stably expressing GFP-H2B.

[**Supplementary**](https://static-content.springer.com/esm/art%3A10.1038%2Fs41467-018-04849-7/MediaObjects/41467_2018_4849_MOESM3_ESM.avi) **Movie 4** Mitotic progression of geldanamycin-treated HeLa cell stably expressing GFP-H2B.


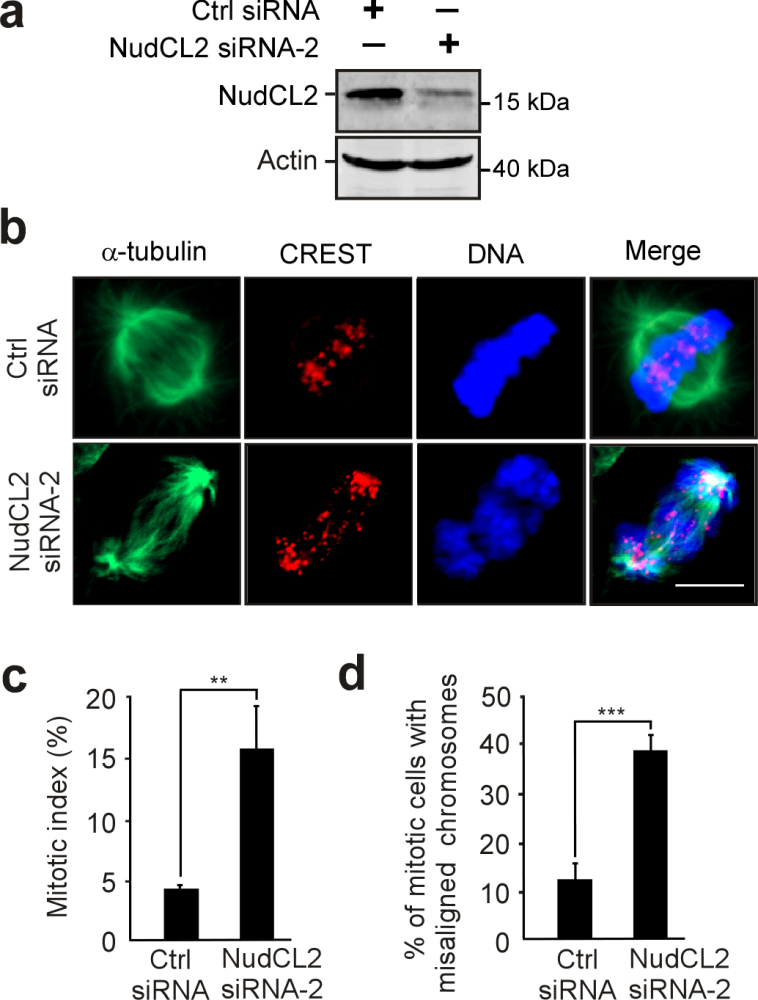


Supplementary Fig. 1


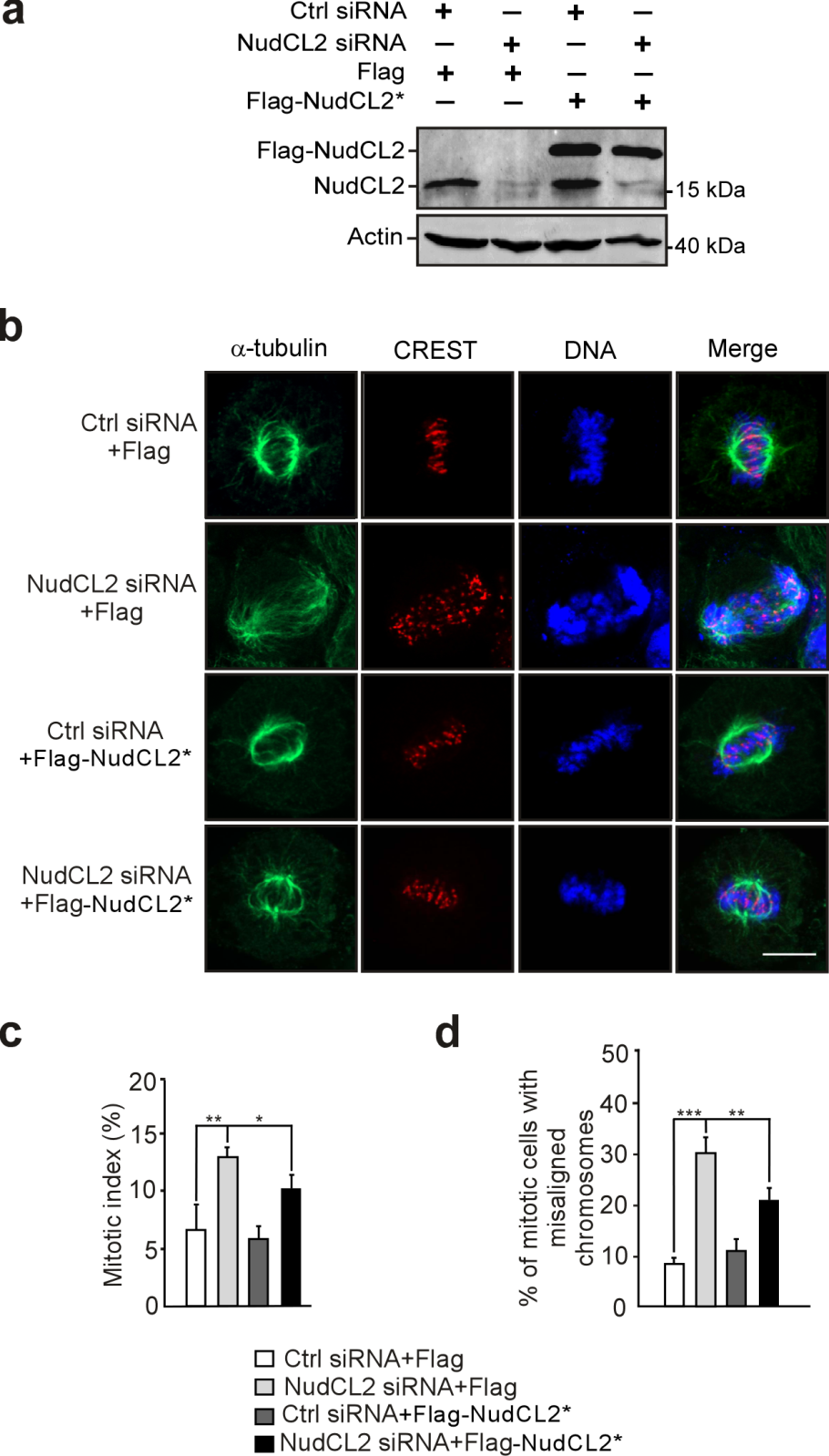


Supplementary Fig. 2


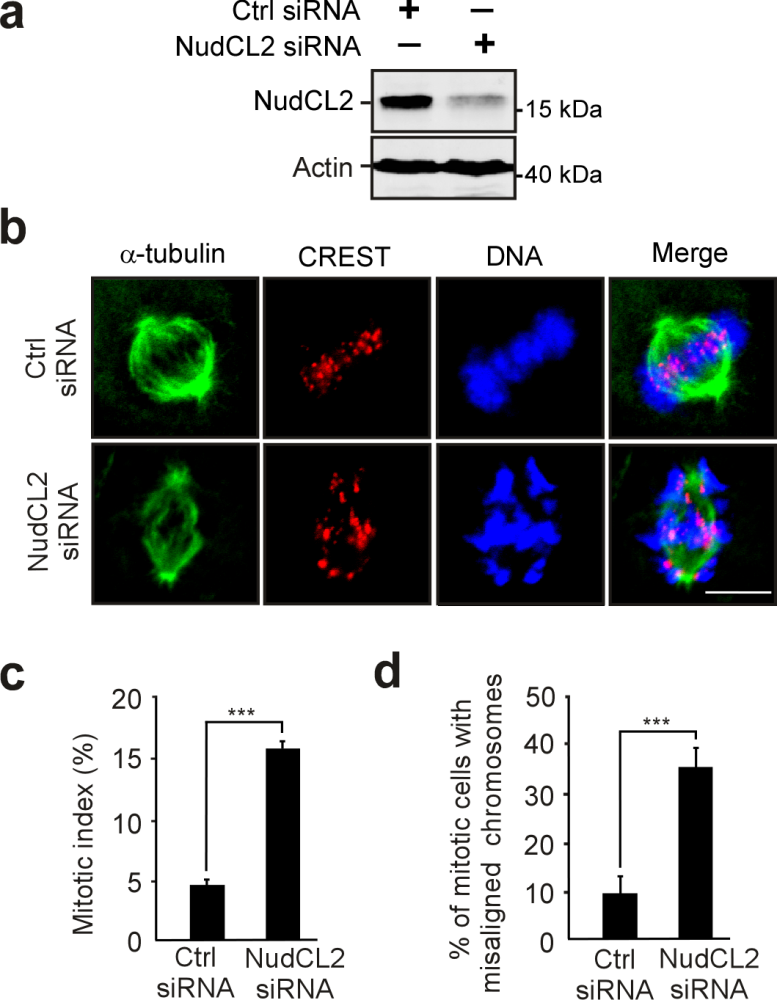


Supplementary Fig. 3

**
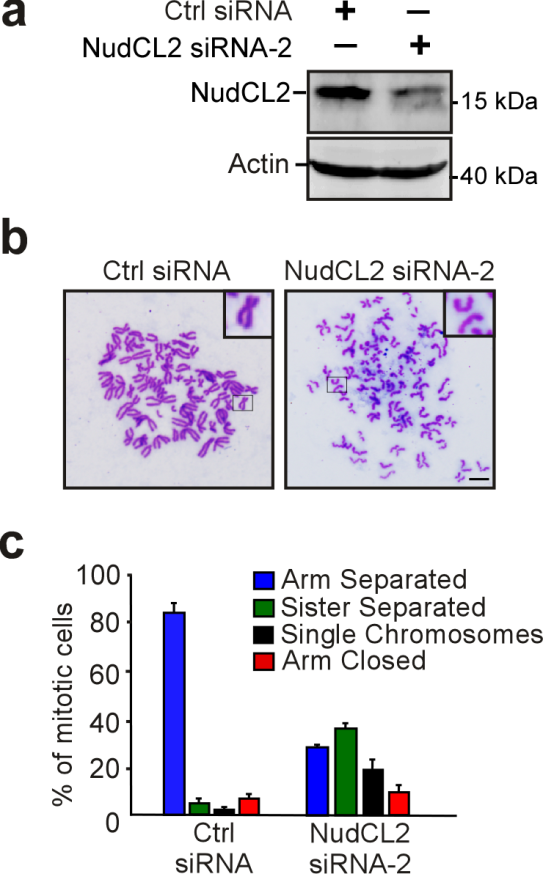
**

Supplementary Fig. 4

**
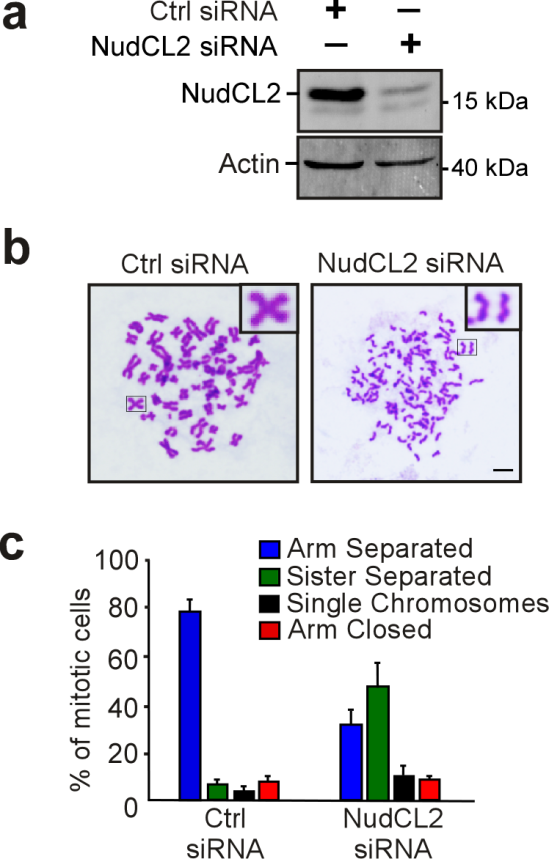
**

Supplementary Fig. 5


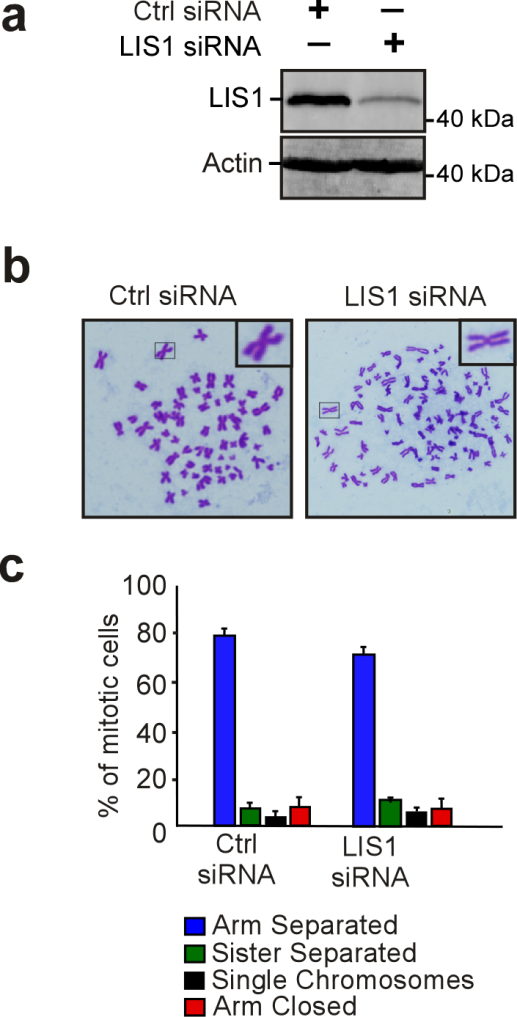


Supplementary Fig. 6


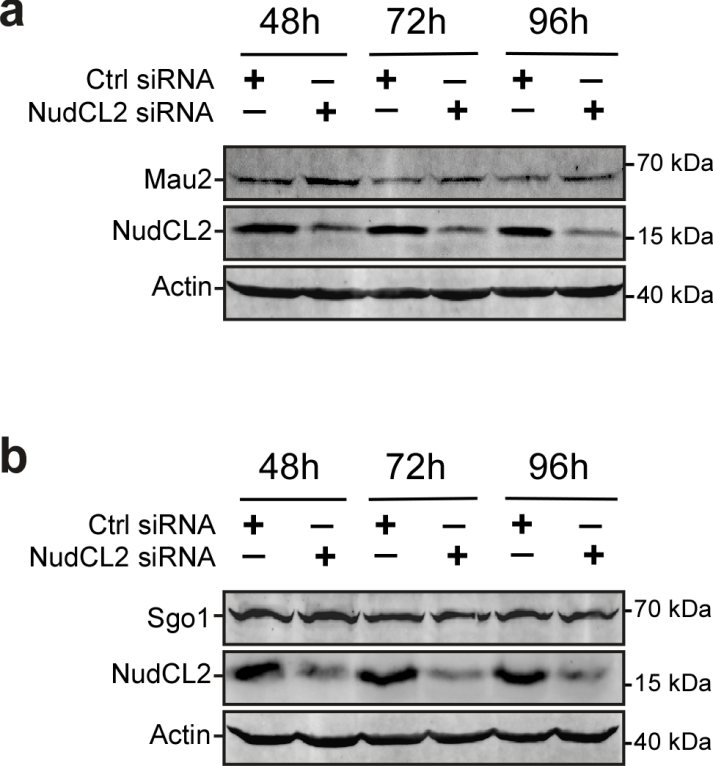


Supplementary Fig. 7


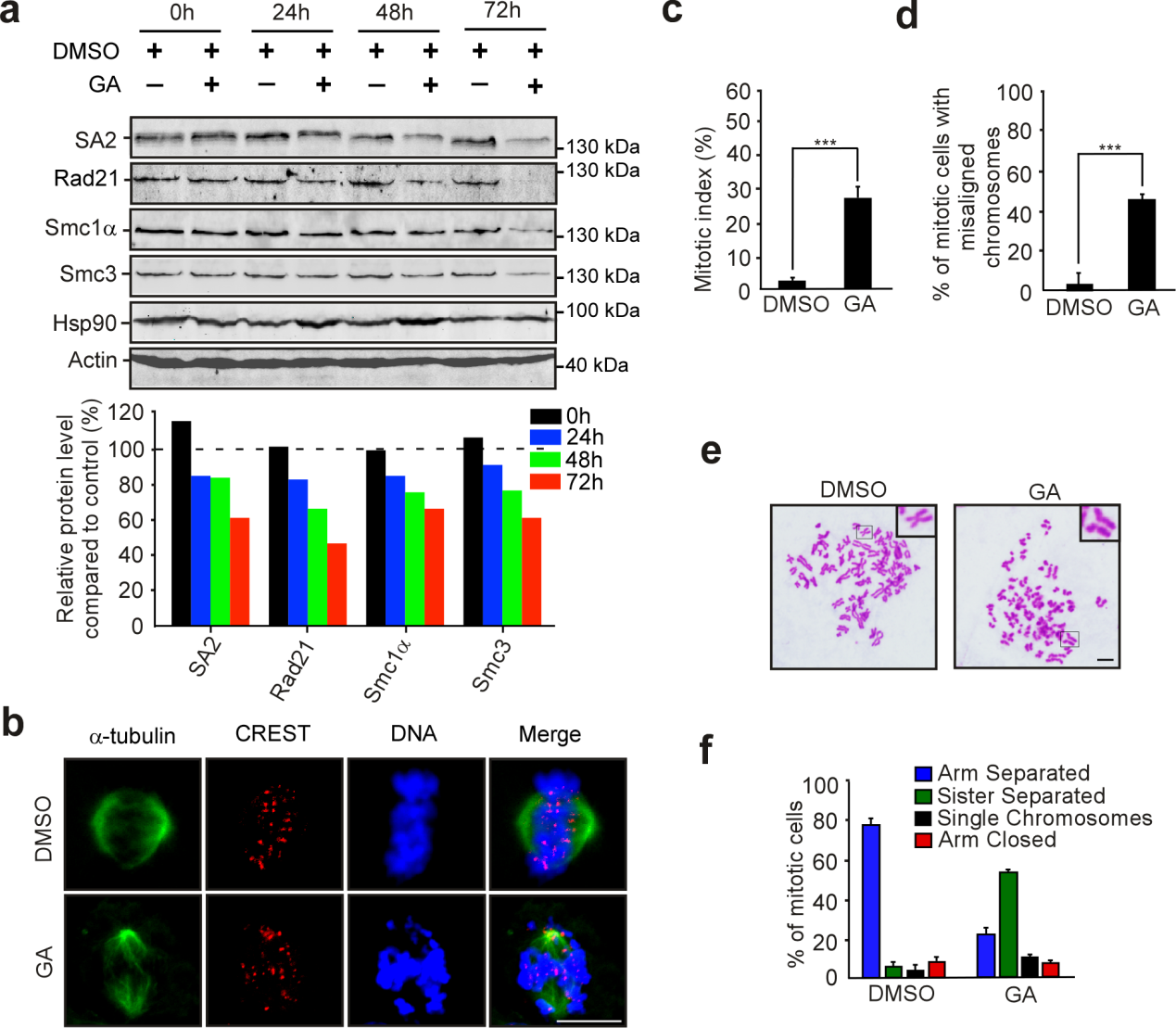


Supplementary Fig. 8


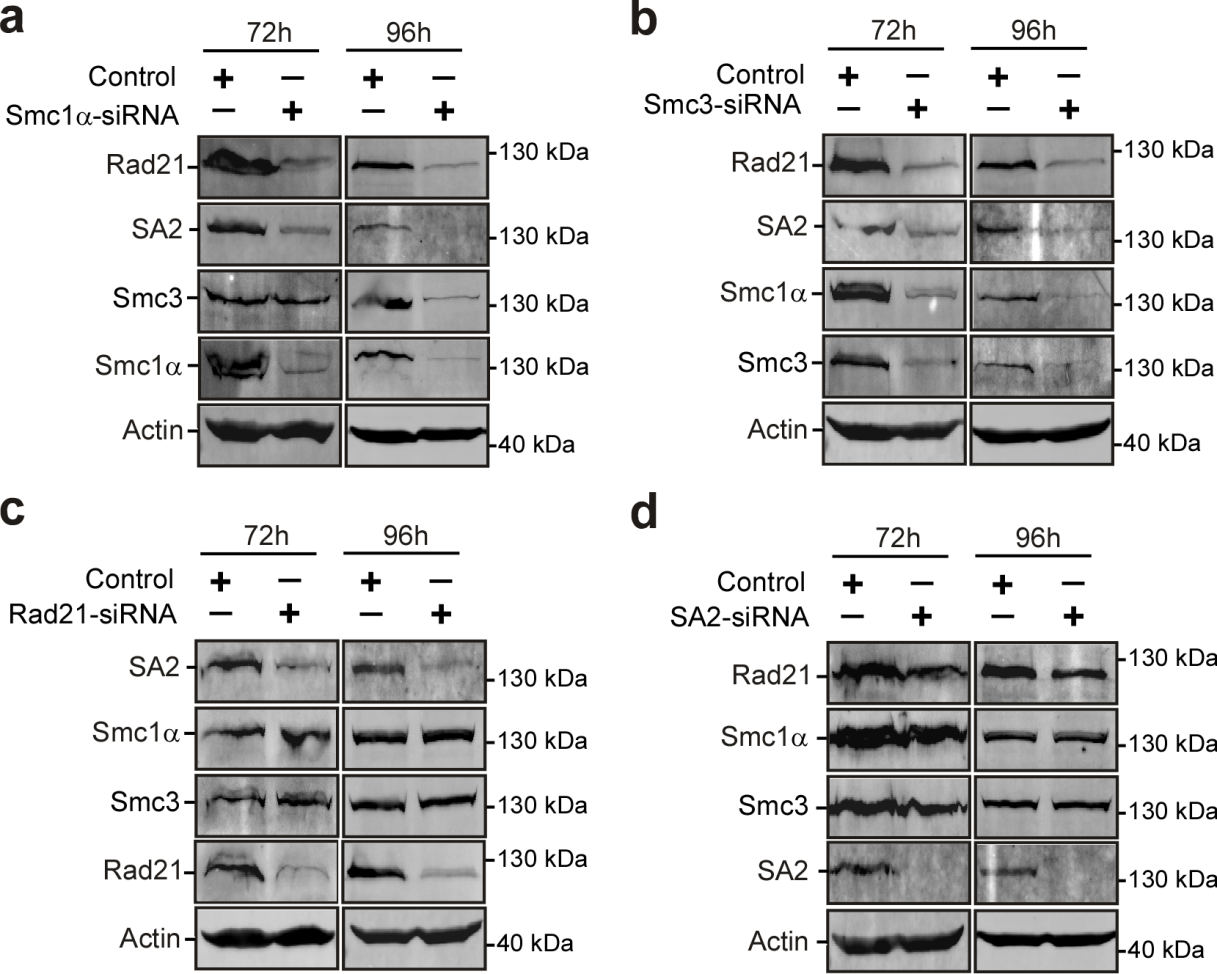


Supplementary Fig. 9
